# Incidence and predictors of LTFU among adults with TB/HIV co-infection in two governmental hospitals, Mekelle, Ethiopia, 2009–2016: survival model approach

**DOI:** 10.1186/s12879-019-3756-2

**Published:** 2019-02-04

**Authors:** Kebede Embaye Gezae, Haftom Temesgen Abebe, Letekirstos Gebreegziabher Gebretsadik

**Affiliations:** Department of Biostatistics, School of Public Health, College of Health Sciences, Mekelle University, P.O. Box: 1871, Mekelle, Ethiopia

**Keywords:** Adults, Cox model, Hazard ratio, LTFU–TB/HIV

## Abstract

**Background:**

Lost to follow-up (LTFU) negatively affects the treatment success of Anti-Retroviral Therapy (ART) and thus, increases Tuberculosis-Human Immunodeficiency Virus (TB/HIV) related morbidity, mortality and hospitalization. However, the incidence and predictors of loss to follow up (LTFU) among adults with TB/HIV co-infection have not yet well-investigated in Ethiopia. Therefore, this study was aimed at investigating the incidence and predictors of LTFU in the study setting in particular.

**Methods:**

A facility based retrospective cohort study was employed among 305 (114 anemic and 191 normal) TB/HIV co-infected adults in two governmental hospitals (Mekelle Hospital and Ayder Comprehensive Specialized Hospital), Mekelle, Ethiopia from 2009 to 2016 and data were collected using checklist. Besides to descriptive statistics, a cox regression analysis was applied to identify statistically significant predictors of LTFU at 5% level of significance. Eventually, the Adjusted Hazard Ratio (AHR) and 95% Confidence Interval (CI) were estimated and interpreted for predictors of LTFU in the final cox model.

**Results:**

Generally, 45 of 305 (14.8%) of TB/HIV co-infected adults were LTFU with an incidence rate of 4.5 new LTFUs per 100 Person Years (PYs) and a median follow up time of 3.1 years (Interquartile Range (IQR): 0.8–5.3 Years). Hemoglobin level ≤ 11.0 g/dl (AHR = 2.660; 95%CI: 1.459–4.848), and any history of OI/s (AHR = 3.795; 95%CI: 1.165–12.364) were risk factors of LTFU. While, adverse drug events (AHR = 0.451; 95%CI: 0.216–0.941), TB treatment completion (AHR = 0.121; 95% CI: 0.057–0.254), and being on Isoniazid Preventive Therapy (IPT) (AHR = 0.085; 95%CI: 0.012–0.628) had protective effect against LTFU.

**Conclusions:**

One in approximately seven TB/HIV co-infected adults had experienced of LTFU with an incidence rate 4.5 LTFUs per 100 PYs. The LTFU rate was higher among adults with low baseline hemoglobin level, no adverse drug events, presence of OI/s, failure to complete TB treatment, and being not on IPT. Therefore, it is advisable to treat anemia and active TB, and preventing the occurrence of OIs including TB using IPT to reduce the incidence of LTFU among TB/HIV co-infected adults.

## Background

From infectious diseases’ statistics, Tuberculosis (TB) and Human Immunodeficiency Virus (HIV) are the leading causes of death globally. Despite many efforts have been made to control and prevent the dual occurrences of the diseases, yet TB is the leading cause of mortality and morbidity in People Living with HIV (PLHIV) [1].

Above one third of the world population is infected with Tuberculosis (TB) and 5–10% of them develop active TB. The reactivation of Latent TB infection (LTBI) to active TB diseases is pronounced among PLHIV by approximately 20 times on average. Globally, approximately one million PLHIV co-infected with TB in 2017. TB is the leading cause of death among PLHIV attributable to 300,000 HIV-associated TB deaths in 2017 in which Africa accounted for the largest shares (84%). Moreover, PLHIV faces the threat of drug-resistant TB. If diagnosis is delayed there is increased risk of mortality from multi-drug resistant and extensively drug-resistant TB [2].

Studies from SSA have also shown that the cumulative incidence of attrition after 3 years of follow-up can be up to 35%. A declined from 86% at 6 months to 77% by 36 months after ART initiation in SSA [3, 4]. Thus, attrition remains a major challenge among adults with HIV and TB in resource limited setting of Low and Middle Income Countries (LMICs) as a result of scarce data on Lost to Follow-Up (LTFU) [5, 6].

Currently, quality of HIV health care services and Anti-Retroviral Therapy (ART) coverage are significantly increasing from time to time. However, LTFU rate is still high and many people drop out at various phases of the treatment pathway. Because of the reasons why people LTFU are not clearly investigated, a poor adherence to treatment is achieved that results in undesired treatment outcomes [7].

LTFU minimizes the benefits of ART in PLHIV which can enhance the occurrence of Opportunistic Infections (OI/s) including TB as a result of immune suppression. Therefore, it intensifies the TB/HIV or Acquired Immune Deficiency Syndrome (AIDS) related negative consequences – morbidity, mortality, and hospitalizations – as a result of dual burdens of the two infections. Moreover, LTFU in HIV or TB/HIV co-infected adults has also adverse consequences such as drug toxicity, treatment failure, and drug resistance that ultimately shorten the survival of patients [8–10].

Different literatures have been shown that LTFU attributes to an increased mortality globally and approximately 1 death every 3 LTFUs (40%) in the SSA region particularly [11–14]. In addition, many studies in the region identified risk factors associated with increased LTFU are mainly: under-nutrition, reduced baseline CD4 count, co-infection with OI/s including active TB, advanced clinical staging, adverse drug reactions, gaps in services, and low access to ART services [3, 13, 15, 16].

Early screening and treatment of TB are on the other hand important to reduce the dual burdens of TB and HIV, reactivation from Latent TB infection (LTBI) to active TB disease, and prevent the transmissions of HIV and TB. Ethiopia has adopted the World Health Organization (WHO) Stop TB strategy which currently is being used as a guideline for the anti TB treatment regimen in almost all health facilities throughout the country since Directly Observed Therapy Short Course (DOTS) full implementation coverage in 2000. Nationally, however, the utilization was yet restricted to approximately 92% due to the low health coverage and poor health access [17].

Generally, LTFU is more common in resource poor setting countries as a result of poor adherence and low health care seeking behavior of people. In an ART in LMICs study, the LTFU after 1 year of ART initiation is greater than 40% that is mainly associated with advanced WHO clinical stage (AIDS) and very low baseline CD4 as a result of late initiation. Though the first 6 months of ART initiation are very important to reduce the LTFU rate, the health care seeking behavior of people remains a major challenge yet to increase retention rate [18, 19].

In Ethiopia, ART was begun in 2003 and free ART was launched in 2005. An estimated 739, 000 Ethiopians are currently living with HIV and all of them eligible to initiate ART irrespective of their baseline CD4 cell count. However, the ART coverage is approximately 76% in adult HIV patients [20]. Despite the high LTFU related negative consequences, little was known even on TB and HIV related LTFU rate in adults in Ethiopia and thus, the incidence and predictors of LTFU are unknown. Thus, this study was aimed at partly filling the gap that will contribute its own role in reducing negative consequences of LTFU among people co-infected with TB and HIV in the country in general and the study setting in particular.

## Methods

### Study setting, design and population

A facility based retrospective cohort study was employed among adults with TB/HIV co-infection who have been initiated ART from 2009 to 2016 at two governmental hospitals (i.e. Mekelle Hospital and Ayder Comprehensive Specialized Hospital), Mekelle, Ethiopia to determine the incidence and predictors of LTFU. Mekelle is the capital city of the national regional state of Tigray which is 783 km far from Addis Ababa, the capital of Ethiopia.

Moreover, Mekelle Hospital has been begun ART services delivery since September, 2004 and up to now, more than 10,000 HIV patients have been followed their ART services. All TB/HIV co-infected patients who have been started ART from January, 2009 to December, 2016 were included in the study for the final analysis. However, TB/HIV patients with unknown ART follow up status and short follow up time (less than 1 month) were excluded from the study.

### Sampling method and sample size determination

In this study, the study subjects were selected from ART registration book based on their TB and HIV status. Furthermore, a minimum sample size was also estimated from previous study that was focused on LTFU conducted in Northern Ethiopia [21] by considering baseline hemoglobin level (anemia status) as an exposure variable (i.e. <= 11.0 g/dl (anemic) as exposed and > 11.0 g/dl (normal) as non-exposed). We used the formula for sample size calculation in survival data analysis based on the following underlining assumptions: two population proportion (q_1_ = proportion exposed group and q_o =_ proportion of non-exposed), 80% statistical power, 95% confidence level, and probability of LTFU from the previous study on LTFU. The number of events (LTFUs) needed to keep the optimum statistical power are estimated to be 62 as follows.

$$ Number\ of\ events\ needed=\frac{{\left({Z}_{\alpha /2}+{Z}_{\beta}\right)}^2}{\log {(HR)}^{2\ast }{q_o}^{\ast }{q}_1}=62 $$

Where: *Z*_*α*/2=_1.96,*Z*_*β*_ = 0.84,Hazard Ratio (HR) = $\frac{Incidence\ of\ LTFU\ among\ exposed\ }{Incidence\ of\ LTFU\ among\ non\ exposed}$ = 0.49

*q*_*o*_ = proportion of non-exposed HIV adults (> 11.0 g/dl) = 57.1% ≈ 0.57

*q*_1_ = proportion of exposed HIV adults (<= 11.0 g/dl) = 42.9% ≈0.43

Probability of LTFU = 9.8% ≈0.10.

However, 305 TB/HIV co-infected adults were included retrospectively from January, 2009 to December, 2016 using all consecutive sampling in the study setting.

### Data collection and study variables

A standardized data collection checklist was prepared by the principal investigator to extract data from the ART registration book and patient medical records (charts). Prior to data collection a pilot test was done and thus, the data collection tool was modified accordingly. The checklist consisted of variables registered during ART initiation and follow up are – Sex, Age, Marital Status, Educational Level, Weight (kg), Body Mass Index (kg/m^2^), Functional Status, WHO clinical stage, Hemoglobin level (g/dl), CD4 Cell Count (cells/mm^3^), Adverse events (Drug Side Effects), IPT prophylaxis, Co-trimoxazole Prophylactic Therapy (CPT), Opportunistic Infections (OI/s) diagnosis, TB Category, Isoniazid Preventive Therapy (IPT), and TB treatment completion – Four trained data collectors were participated in data collection. In addition, data collectors were supervised and data collection tool (checklist) was assessed for completeness, consistency and accuracy by the principal investigator through daily supervision.

The ART follow up status was recorded as dead, alive, transfer, LTFU, drop out or stop. Patients who missed their ART follow up for at least three consecutive months recorded as LTFU. The event was LTFU and the outcome variable was time to LTFU. Thus, the event of interest (LTFU) was labeled as 1 and 0 else considered as censored.

### Statistical data analysis

First, data were entered, and then exported to STATA version 12.0. Data were cleaned and categorical variables were labeled prior to data analysis and data were also declared to be survival-time data. Survival data analysis was done using cox regression analysis besides to the descriptive statistics. Kaplan Meier (KM) curves were drawn to estimate the probability of LTFU and proportionality hazards assumption was checked for covariates intended to be included in the final cox model. Log rank test was used to select categorical predictors. Decision was based on a *p*-value of 0.25 in univariate analysis for potential candidate variables selection to be considered in the final model. Variables with a p-value of less than 0.05 (5%) were considered as statistically significant predictors of LTFU after interaction effects and model diagnostics checked. Finally, the Adjusted Hazard Ratio and 95% CI were interpreted for the statistically significant predictors of LTFU in the final cox model.

## Results

### Baseline socio demographic characteristics and LTFU rate of adults with TB/HIV co-infection

A total of 305 TB/HIV co-infected adults were involved in the study that contributes 944.4 PYs. Above half (51.5%) of the study subjects were females, and above two –fifth (42.3%) of them had also an age above their median (> 35 years), 129 (42.3%) had married marital status, 119 (39.0%) had secondary educational status.

Regarding LTFU, 45 of 305 (14.8%) of adults co-infected with TB/HIV experienced LTFU throughout the entire follow up time and the overall LTFU rate was found to be approximately 4.5 LTFU cases per 100 PYs with a median follow up time of 3.1 years (Interquartile Range (IQR): 0.8–5.3 Years). Moreover, 26 of 45 (57.8%) of the LTFU cases were experienced among females with an incidence rate of 5.1 new cases per 100 PYs. On the other hand, majority (68.9%) of the total LTFUs were also seen among adults who had an age above the median (> 35 years) and approximately 89.0% of the event of interest was observed among orthodox followers (Table 1).

**Table 1:** Baseline socio-demographic characteristics and distribution by LTFU status of adults with TB/HIV co-infection in two governmental hospitals, Mekelle, Ethiopia, 2009–2016 (*n* = 305)

Socio-demographic characteristics	LTFU (*n*_1_ = 45) Frequency (%)	No-LTFU (*n*_2_ = 260) Frequency (%)	Total (*n* = 305) Frequency (%)	LTFU Rate [95%CI] Per 100 PYs	*P*-value
Sex					0.3644
Male	19 (42.2)	129 (49.6)	148 (48.5)	3.9 [2.5, 6.1]	
Female	26 (57.8)	131 (50.4)	157 (51.5)	5.1 [3.5, 7.5]	
Age (Years)					0.1078
> 35	14 (31.1)	115 (44.2)	129 (42.3)	3.3 [1.9, 5.5]	
≤ 35	31 (68.9)	145 (55.8)	176 (57.7)	5.5 [3.9, 7.8]	
Marital Status					0.3710
Single	13 (28.9)	56 (21.6)	69 (22.6)	6.2 [3.6, 10.7]	
Married	20 (44.4)	109 (41.9)	129 (42.3)	4.5 [2.9, 7.0]	
Others^a^	12 (26.7)	95 (36.5)	107 (35.1)	3.5 [2.0, 6.1]	
Educational Level					0.5581
No Education	10 (22.2)	55 (21.2)	65 (21.3)	4.4 [2.4, 8.1]	
Primary	11 (24.4	79 (30.4)	90 (29.5)	3.8 [2.1, 6.8]	
Secondary	21 (46.7)	98 (37.7)	119 (39.0)	5.7 [3.7, 8.7]	
Tertiary	3 (6.7)	28 (10.8)	31 (10.2)	2.8 [0.9, 8.7]	
Religion					0.0077
Orthodox	40 (88.9)	253 (97.3)	293 (96.1)	4.1 [3.0, 5.6]	
Muslim	5 (11.1)	7 (2.7)	12(3.9)	21.1 [8.8, 50.8]	
Health Institution					0.6074
Ayder Hospital	21 (46.7)	112 (43.1)	133(43.6)	5.0 [3.3, 7.7]	
Mekelle Hospital	24 (52.3)	148 (56.9)	172(56.4)	4.2[2.8, 6.2]	

Others^a^: Divorced/Widowed/Separated

### Baseline clinical characteristics and distribution by LTFU status of adults with TB/HIV co-infection

Based on their baseline clinical characteristics, above half (51.5%) of the TB/HIV adults had a baseline BMI above the median (BMI > 17.4 kg/m^2^). In addition, 54 of 305 (17.7%), 142 of 305 (46.6%), 250 of 305 (82.0%), and 114 of 305 (37.4%) of adults had baseline: bedridden functional status, clinical stage IV, CD4 cell count (<=200 cells/mm^3^), and anemic status, respectively.

Based on their LTFU status, 62.2% of the total LTFU cases were seen in TB/HIV adults with a baseline BMI of <=17.4 kg/ m^2^ with an incidence rate of 6.6 new cases per 100 PYs and on the other hand the LTFU rate was 8.5 per 100 PYs among TB/HIV adults with baseline anemic status (Table 2).

**Table 2:** Baseline clinical characteristics and distribution by LTFU status of adults with TB/HIV co-infection in two governmental hospitals, Mekelle, Ethiopia, 2009–2016 (*n* = 305)

Baseline characteristics	LTFU (*n*_1_ = 45) Frequency (%)	No-LTFU (*n*_2_ = 260) Frequency (%)	Total (*n* = 305) Frequency (%)	LTFU Rate [95%CI] Per 100 PYs	*P*-value
Body Mass Index (kg/m^2)^					0.0138
> 17.4	17 (37.8)	131 (50.4)	148 (48.5)	3.0 [1.8, 4.8]	
< =17.4	28 (62.2)	129 (49.6)	157 (51.5)	6.6 [4.6, 9.6]	
Functional Status					0.1476
Working	17 (37.8)	124 (47.7)	141 (46.2)	3.2 [2.0, 5.2]	
Ambulatory	19 (42.2)	91 (35.0)	110 (36.1)	5.4 [3.5, 8.5]	
Bedridden	9 (20.0)	45 (17.3)	54 (17.7)	7.8 [3.4, 14.2]	
Clinical Stage					0.1093
I or II	4 (8.9)	28 (10.8)	32 (10.5)	3.2 [1.2, 8.6]	
III	15 (33.3)	116 (44.6)	131 (42.9)	3.3 [2.0, 5.4]	
IV	26 (57.8)	116 (44.6)	142 (46.6)	6.3 [4.3, 9.3]	
CD4 Count (Cells/mm^3^)					0.6213
> 200	8 (17.8)	47 (18.1)	55 (18.0)	3.8 [1.9, 7.6]	
< =200	37 (82.2)	213 (81.9)	250 (82.0)	4.7 [3.4, 6.5]	
Hemoglobin level (g/dl)					0.0002
Normal (> 11.0)	19 (42.2)	172 (66.2)	191 (62.6)	2.8 [1.8, 4.3]	
Anemic (<=11.0)	26 (57.8)	88 (33.8)	114 (37.4)	8.5 [5.8, 12.5]	

**Fig. 1:**
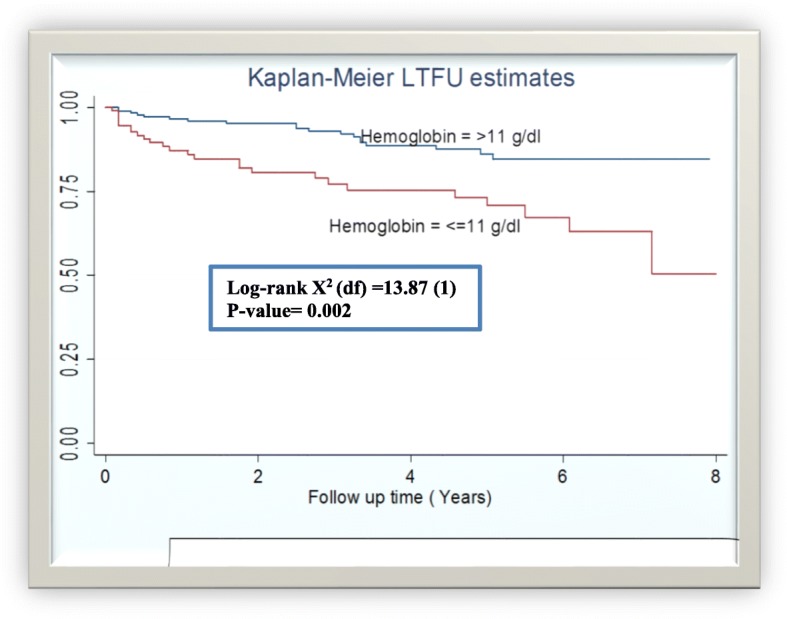
LTFU estimates of adults with TB/HIV co-infection by baseline hemoglobin level in two governmental hospitals, Mekelle, Ethiopia, 2009–2016 (*n* = 305)

Moreover, when we draw KM curve based on baseline hemoglobin level to compare the LTFU estimates – It was higher among the anemic TB/HIV co-infected adults than their normal counter parts (Fig. 1).

### Diagnosis and treatment characteristics and distribution by LTFU status of adults with TB/HIV co-infection

Based on this category, approximately 16.0% of HIV patients were under IPT during the course of their follow up and 83.3% were taken CPT as a means to prevent OI/s. To the contrary, majority (63.0%) of TB/HIV co-infected adults were not reported any adverse drug events with a LTFU rate of approximately twice those we had been experienced with adverse events (Table 3).

**Table 3:** Diagnosis and treatment characteristics and distribution by LTFU status of adults with TB/HIV co-infection in two governmental hospitals, Mekelle, Ethiopia, 2009–2016 (*n* = 305)

Diagnosis and Treatment characteristics	LTFU (*n*_1_ = 45) Frequency (%)	No-LTFU (*n*_2_ = 260) Frequency (%)	Total (*n* = 305) Frequency (%)	LTFU Rate [95%CI] Per 100 PYs	*P*-value
Adverse drug events					0.0087
No	39 (86.7)	156 (60.0)	192 (63.0)	6.0 [4.4, 8.4]	
Yes	6 (13.3)	104 (40.0)	113 (37.0)	3.3 [1.2, 4.3]	
History OI/s					0.0180
No	3 (6.7)	59 (22.7)	62 (20.3)	1.4 [0.5, 4.5]	
Yes	42 (93.3)	201 (77.3)	243 (79.7)	5.3 [3.9, 7.2]	
TB Category					0.0530
PTB	16 (35.6)	116 (44.6)	132 (43.3)	3.1 [1.9, 5.1]	
EPTB/Mixed	29 (64.4)	144 (55.4)	173 (56.7)	6.0 [4.2, 8.7]	
TB treatment completion					< 0.001
No	13 (28.9)	39 (15.0)	52 (17.0)	35.7 [20.7, 61.5]	
Yes	32 (71.1)	221 (85.0)	253 (83.0)	3.3 [2.4, 4.7]	
IPT					0.0025
on	1 (2.2)	47 (18.1)	48 (15.7)	5.6 [4.2, 7.6]	
off	44 (97.8)	213 (81.9)	257 (84.3)	0.5 [0.1, 3.3]	
CPT					0.9189
Off	7 (15.6)	44 (16.9)	51 (16.7)	4.7 [2.2, 9.8]	
On	38 (84.4)	216 (83.1)	254 (83.3)	4.5 [3.3, 6.2]	

*OI/s* opportunistic Infection/s, *PTB* Pulmonary Tuberculosis, *EPTB* Extra Pulmonary Tuberculosis, *CPT* Co-trimoxazole Prophylactic Therapy, *IPT* Isoniazid Preventive Therapy

### Predictors of LTFU among adults with TB/HIV co-infection in the final cox model

Five variables became statistically significant in the final cox model: Baseline hemoglobin level (AHR = 2.660; 95% CI: 1.459–4.848), adverse drug events (AHR = 0.451; 95%CI: 0.216–0.941), history of OI/s (AHR = 3.795; 95%CI: 1.165–12.364), TB Treatment Completion (AHR = 0.121; 95%CI: 0.057–0.254), and being on IPT (AHR = 0.085; 95%CI: 0.012–0.628). Thus, TB/HIV co-infected patients with anemia status, and presence of at least one OI were approximately 2.66, and 3.80 times more likely to LTFU than their respective comparative groups, respectively. However, TB/HIV co-infected patients who: reported adverse drug events, completed TB treatment phase, and being on IPT were protected by nearly – 55%, 88%, and 92% - against LTFU than their counter reference categories, respectively (Table 4).

**Table 4:** Statistically significant predictors of LTFU among adults with TB/HIV co-infection in two governmental hospitals, Mekelle, Ethiopia, 2009–2016 (*n* = 305)

Variables	CHR ([95% CI)	AHR [95% CI]	*P*-Value
Hemoglobin			
> 11(Normal)	1.000	1.000	
≤ 11(Anemic)	2.928 [1.617, 5.304]	2.660 [1.459, 4.848]	0.001^**^
Adverse drug events			
No	1.000	1.000	
Yes	0.390 [0.188, 0.811]	0.451[0.216, 0.941]	0.034^*^
History of OI			
No	1.000	1.000	
Yes	3.720 [1.153, 12.008]	3.795 [1.165, 12.364]	0.027^*^
TB Treatment Completion			
No	1.000	1.000	
Yes	0.121 [0.060, 0.244]	0.121[0.057, 0.254]	< 0.001^**^
IPT			
Off	1.000	1.000	
On	0.089 [0.012, 0.645]	0.085 [0.012, 0.628]	0.016^*^

1.000: Reference Category: *CHR* Crude Hazard Ratio, *AHR* Adjusted Hazard Ratio, *CI* Confidence interval

^*^Significant at 5% level of significance,^**^significant at 0.1% level of significance (α)

## Discussion

The current study was investigated to determine the incidence and predictors of LTFU among adults with TB/HIV co-infection in the study setting. Thus, the incidence rate was found to be 4.5 new LTFU cases per 100 PYs with a median follow up time of 3.1 years (IQR: 0.8–5.3 years). Up on running our final cox model, statistically significant predictors of LTFU are: baseline hemoglobin level, adverse drug events, history of OI/s, TB treatment completion, and IPT (Table 4**)**.

In the current study, 45 of 305 (14.8%) TB/HIV co-infected patients developed the event of interest (LTFU). The incidence rate was found to be 4.5 per 100 PYs collectively among TB/HIV co-infected adults. The incidence of LTFU was lower as compared to other studies conducted in India, Vietnam, South Africa (Johannesburg), and SSA region [3, 11, 22, 23]. To the contrary, the LTFU rate was higher as compared to other studies conducted in Vietnam, South Africa, Cameroon, Zambia, and Liberia [24–28]. However, the LTFU rate is similar to a prospective study conducted in Ethiopia [29] . The high incidence rate in the current study may be due to the duel burden of TB and HIV might be triggered the risk of LTFU as compared to the majority of other studies in which their study subjects were HIV or TB patients alone. Thus, this finding strengthens the idea that LTFU remains a major challenge among adults with TB and HIV in resource limited setting of LMICs such as Ethiopia as a result of scarce data [5, 6] and retention of patients in care remains a major challenge to the health care providers and program mangers [18, 19].

In this study, LTFU was significantly affected by the baseline hemoglobin level where the incidence of LTFU is roughly twice among the anemic groups than their normal counterpart (AHR = 2.660; 95%CI: 1.459–4.848**)**. Finding of the current study is consistent with a study conducted in Myanmar, India though that has estimated Odds Ratio (OR) instead of HR and the study subjects were HIV patients than TB/HIV co-infected [30]. However, another study that has been done in Central Tigray, Ethiopia reported the non-predictive effect of baseline hemoglobin to LTFU among HIV Patients under ART [21]. In fact, multiple medications for TB, HIV and Anemia as well may lead to LTFU of the patient from ART follow up due to multiple burdens.

Moreover, patients who have been reported any adverse drug events during the course of their treatment were experienced low risk of LTFU (AHR = 0.451; 95% CI: 0.216–0.941). Though literatures on this study variable were scarce, a facility based case control study has been revealed that presence of drug side effect among HIV/AIDS patient was significant risk factor of LTFU in which patients with adverse drug side effect had approximately 12 times the odds of developing LTFU than those in the control group [31]. The unusual findings observed in this study can be seen from the side that the high health care seeking behavior of the patients may contribute for high adherence to treatment and thus, they might report adverse events if they were experienced which in turn leads to replacement by the safest medication by their doctors’ that may tend to decrease the LTFU rate. On the other hand, patients who did not report any drug side effects might be negligent about the adverse effects irrespective of the consequences of LTFU such as morbidity, hospitalization and eventually death. Moreover, the degree of Scaling up LTBI treatment using IPT was higher among those with adverse drug events that could possibly both increase adherence and decrease LTFU related mortality among adults (18.6% IPT coverage among those with adverse events as compared to 14.1% among those did not report it).

Any history of OI/s was also statistically positively associated with LTFU among TB/HIV co-infected adults. Controlling the effect of other predictors, TB/HIV adults who have been diagnosed with at least one OI during the course of their treatment were approximately 4 times more likely to experience LTFU than those who did not (Table 4). It is possibly justifiable that the multiple burdens of diseases may affect the psychological curability and diminish the immune status of the patient and eventually which may result in LTFU from their regular course of treatment.

Successfully completing TB treatment had also a protective effect over LTFU among TB/HIV co-infected adults (AHR =0.121; 95% CI: 0.057–0.254). It is evidenced that both TB and HIV have bidirectional and synergistic relation-ships and thus, the presence of either infection has its own significant contribution for the occurrence of other. The strong association between the two infections will be broken up on the successful completion of TB treatment that may reduce the dual burdens proportionally through TB treatment success rate. Therefore, the health status would be improved, and the incidence of LTFU on the other hand will be decreased as a result of treatment success for TB.

In the current study, majority (84.3%) of the patients were off IPT prophylaxis (Table 3). Regarding the statistical association, on IPT adults had low risk of LTFU than those of off IPT one (Table 4). The finding of the current study is consistent with a facility based retrospective cohort and follow up studies, and another case control study conducted in Mizan Aman, Southern Ethiopia, and Wukro (all in Ethiopia), respectively that have been revealed all off IPT is a potential risk factor of LTFU among HIV patients [31–33]. This could be related to preventing LTBI reactivation by IPT is simpler than treating active TB disease using full regiment of TB treatment so that people tend to LTFU as a result of dual burdens of both diseases and dual follow ups as well.

The cause and effect relationship of TB and HIV (was TB or HIV come first) is in fact unclear and considered as TB/HIV co-infected regardless of their temporal relationship. Literatures are also scarce and majority of the literatures are LTFU about HIV patients than TB/HIV co-infected. Moreover, some HIV adults who were on IPT initially tend to develop active TB and thus, started anti TB regimen that may mislead to that IPT was given to active TB co-infected adults.

## Conclusions

Overall, 1 in approximately seven of TB/HIV co-infected adults had experienced of LTFU with an incidence rate 4.5 LTFUs per 100 PYs. TB/HIV co-infected adults diagnosed with anemia at base line, and diagnosed with any OI/s have been strengthened the occurrence of LTFU. Conversely, being on IPT, Completing TB treatment, and developing adverse drug events had protective effect against LTFU among TB/HIV co-infected adults. Therefore, it is advisable to treat anemia and active TB, and preventing the occurrence of OIs including reactivation of latent TB using IPT to reduce the incidence of LTFU among TB/HIV co-infected adults.

## Abbreviations

AHR: Adjusted Hazard Ratio
AIDS: Acquired Immunodeficiency Virus
ART: Anti-Retroviral Treatment
CI: Confidence Interval
CPT: Co-trimoxazole Prophylactic Therapy
DOTS: Directly Observed Therapy Short-course
HIV: Human Immunodeficiency Virus
IPT: Isoniazid Preventive Therapy
IQR: Interquartile Range
KM: Kaplan Meier
LMIC: Low and Middle Income Countries
LTBI: Latent Tuberculosis Infection
LTFU: Lost to Follow-up
OI: Opportunistic Infection
PYs: Person Years
SSA: Sub Saharan Africa
WHO: World Health Organization
